# 2,6-Bis(4-meth­oxy­phen­yl)-1,4-dithiine

**DOI:** 10.1107/S1600536814000397

**Published:** 2014-01-15

**Authors:** Sha-Sha Zhao, Qiong Su, Zhi-Hong Peng, De-Lie An

**Affiliations:** a Department of Chemistry, College of Chemistry and Chemical Engineering, Hunan University, Changsha 410082, People’s Republic of China

## Abstract

The title mol­ecule, C_18_H_16_O_2_S_2_, reveals crystallographic twofold rotation symmetry (with both S atoms lying on the axis) and one half-mol­ecule defines an asymmetric unit. The dithiine ring is in a boat conformation. The aromatic ring and the C=C bond are nearly coplanar, with small torsion angles of −171.26 (19) and 8.5 (3)°. The two S—C bond lengths [1.7391 (19) and 1.7795 (18) Å] are shorter than single C—S bonds and longer than analogous C=S double bonds, which indicates a certain degree of conjugation between the lone pair on the S atom and π electrons of the C=C bond. The crystal packing only features van der Waals inter­actions.

## Related literature   

For a similar crystal structure, 2,6-diphenyl-1,4-dithiine, see: Piao *et al.* (2004 ▶). For background to 1,4-dithiine derivatives, see: Kobayashi & Gajurel (1986 ▶); Scott *et al.* (2000 ▶). For the synthesis of a similar compound, see: Nakayama *et al.* (1984 ▶). For standard bond lengths, see: Allen *et al.* (1987 ▶).
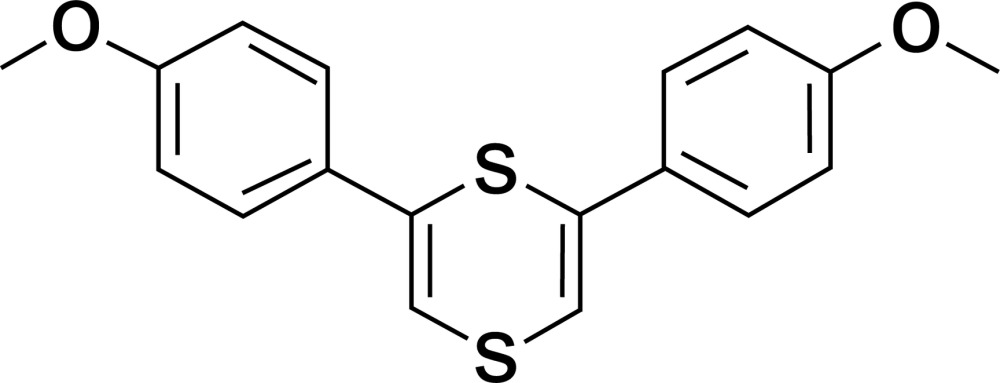


## Experimental   

### 

#### Crystal data  

C_18_H_16_O_2_S_2_
*M* *_r_* = 328.43
Orthorhombic, 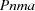

*a* = 10.1330 (11) Å
*b* = 27.318 (3) Å
*c* = 5.5402 (6) Å
*V* = 1533.6 (3) Å^3^
*Z* = 4
Mo *K*α radiation
μ = 0.35 mm^−1^
*T* = 293 K
0.21 × 0.18 × 0.09 mm

#### Data collection  

Bruker SMART CCD area-detector diffractometer
Absorption correction: multi-scan (*SADABS*; Bruker, 2001 ▶) *T*_min_ = 0.121, *T*_max_ = 1.000
8513 measured reflections
1541 independent reflections
1258 reflections with *I* > 2σ(*I*)
*R*_int_ = 0.043

#### Refinement  

*R*[*F* ^2^ > 2σ(*F* ^2^)] = 0.038
*wR*(*F* ^2^) = 0.102
*S* = 1.04
1541 reflections
104 parameters
H-atom parameters constrained
Δρ_max_ = 0.29 e Å^−3^
Δρ_min_ = −0.16 e Å^−3^

### 

Data collection: *SMART* (Bruker, 2001 ▶); cell refinement: *SAINT* (Bruker, 2001 ▶); data reduction: *SAINT*; program(s) used to solve structure: *SHELXS97* (Sheldrick, 2008 ▶); program(s) used to refine structure: *SHELXL97* (Sheldrick, 2008 ▶); molecular graphics: *SHELXTL* (Sheldrick, 2008 ▶); software used to prepare material for publication: *SHELXTL*.

## Supplementary Material

Crystal structure: contains datablock(s) I, New_Global_Publ_Block. DOI: 10.1107/S1600536814000397/kp2462sup1.cif

Structure factors: contains datablock(s) I. DOI: 10.1107/S1600536814000397/kp2462Isup2.hkl

Supporting information file. DOI: 10.1107/S1600536814000397/kp2462Isup3.cml

CCDC reference:

Additional supporting information:  crystallographic information; 3D view; checkCIF report

## Figures and Tables

**Fig. 1.**
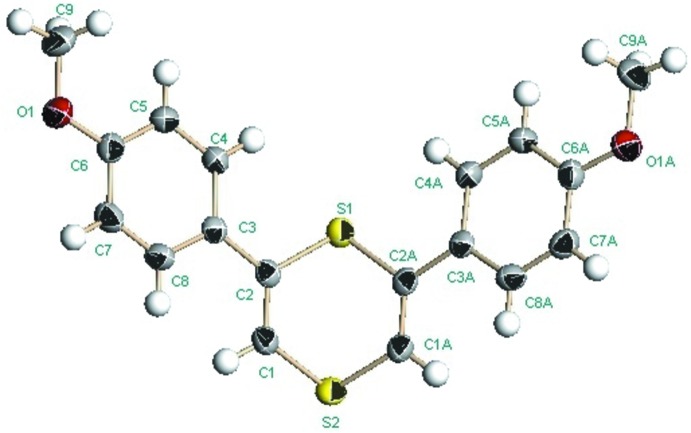
Structural unit of the title molecule with atom labelling scheme and 50% probability displacement ellipsoids. Symmetry code to generate the entire molecule:x,-y+1/2,z.

**Fig. 2.**
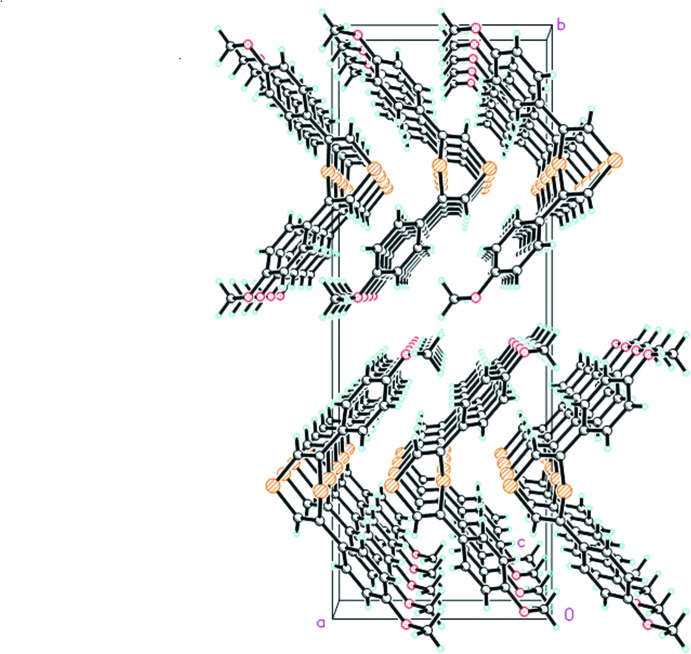
Crystal packing reveals a columns of molecules held together by van der Waals interactions, only.

## supplementary crystallographic information

### 1. Comment

1,4-Dithiine derivatives are very important intermediates in organic synthesis and can be used as versatile building blocks for a variety of chemical purposes (Kobayashi & Gajurel, 1986). In addition, some 1,4-dithiine derivatives have exhibited good biological activities, For example, Scott *et al.* showed that 2,3-dihydro-2-phenyl-1,4-dithiin-1,1,4,4-tetroxide could be used as nonpeptide antagonist of the human Galanin hGAL-1 receptor (Scott *et al.*, 2000). Unfortunately, there are very few acceptable methods to prepare 1,4-dithiine compounds thus far, and, in most cases, a successful protocol must use bis(arylethanonyl) sulfides compounds as precursors. Herein we report a new synthetic approaches and crystal structure of 2,6-bis(4-methoxyphenyl)-1,4-dithiine.

The molecular structure of the title compound(I) (Fig. 1) exhibits a twofold rotation axes symmetry. The dithiine ring is in a boat conformation. In the crystal, dominate columns of assembled molecules, however, their separation distances are larger than 5.5402 (13) Å (Fig. 2). The bond lengths of C1—C2 in heterocyclic ring presents a characteristic of the C=C double bond. An aromatic ring and the C=C bond are nearly coplanar, with small torsion angles of -171.26 (19)° and 8.5 (3)° for C1—C2—C3—C4 and C1—C2—C3—C8,respectively ·. The two characteristic bond lengths of S1—C2 and S2—C1 are shorter than C—S single bonds and longer than analogous C=S double bonds (Allen *et al.*, 1987), which indicates a certain degree of conjugation between the lone pair on the sulfur atom and π electrons of the C=C bond.

### 2. Experimental

NaOEt (224 mg, 3.3 mmol) was dissolved in alcohol (10 mL), and then added to bis(4-methoxyphenylethynyl) sulfide (97.8 mg,0.33 mmol). After the mixture was stirred at room temperature for 10 min, Na_2_S·9H_2_O (159 mg, 0.66 mmol) was added. The resulting mixture was then stirred at reflux temperature for 2 h. The reaction mixture was cooled to room temperature and quenched by water and extracted with dichloromethane. The extract was then washed with brine, dried over Na_2_SO_4_, and filtered. The solvent was evaporated *in vacuo*, and the residue was chromatographed (SiO_2_; eluent, ether/dichloromethane, 4: 1) to give 93 mg of compound I (85%) as a yellow solid: mp 401–403 K; ^1^H NMR (400 MHz, CDCl_3_): *δ* = 3.83 (s, 6H; OCH_3_), 6.42 (s, 2H; CH), 6.89(d, *J* = 8.8 Hz, 4H; Ar—H), 7.58 (d, *J* = 8.8 Hz, 4H; Ar—H); ^13^C NMR (100 MHz, CDCl_3_): *δ* = 55.50 (OCH_3_), 114.07 (CH), 116.40 (CH), 128.45 (CH), 129.83 (C), 139.52 (C), 160.10 (C); IR (KBr): *ν* = 3020, 2959, 2914, 1607, 1508, 1457, 1258, 1191, 832 cm^-1^; MS (EI): *m/z* (%): 328.2 (*M*^+^, 100), 313.1 (43); HRMS (EI): *m/z* Calcd for C_18_H_16_O_2_S_2_ 328.0584, Found 328.0586.

Single crystals of (I) suitable for X-ray diffraction analysis was obtained by slow diffusion of petroleum ether into a dichloromethane solution at 298 K.

### 3. Refinement

H atoms were refined with fixed individual displacement parameters [*U*_iso_ (H) = 1.2 *U*_eq_ (C) and *U*_iso_ (H) = 1.5 *U*_eq_ (C methyl)] using a riding model, with aromatic C—H = 0.93 Å, methyl C—H = 0.96 Å.

### Crystal data

**Table:**

C_18_H_16_O_2_S_2_	*F*(000) = 688
*M**_r_* = 328.43	*D*_x_ = 1.422 Mg m^−^^3^
Orthorhombic, *P**n**m**a*	Mo *K*α radiation, λ = 0.71073 Å
Hall symbol: -P 2ac 2n	Cell parameters from 1658 reflections
*a* = 10.1330 (11) Å	θ = 7.5–53.8°
*b* = 27.318 (3) Å	µ = 0.35 mm^−^^1^
*c* = 5.5402 (6) Å	*T* = 293 K
*V* = 1533.6 (3) Å^3^	Prismatic, yellow
*Z* = 4	0.21 × 0.18 × 0.09 mm

### Data collection

**Table:**

Bruker SMART CCD area-detector diffractometer	1541 independent reflections
Radiation source: fine-focus sealed tube	1258 reflections with *I* > 2σ(*I*)
Graphite monochromator	*R*_int_ = 0.043
phi and ω scans	θ_max_ = 26.0°, θ_min_ = 3.0°
Absorption correction: multi-scan (*SADABS*; Bruker, 2001)	*h* = −12→12
*T*_min_ = 0.121, *T*_max_ = 1.000	*k* = −33→32
8513 measured reflections	*l* = −5→6

### Refinement

**Table:**

Refinement on *F*^2^	Primary atom site location: structure-invariant direct methods
Least-squares matrix: full	Secondary atom site location: difference Fourier map
*R*[*F*^2^ > 2σ(*F*^2^)] = 0.038	Hydrogen site location: inferred from neighbouring sites
*wR*(*F*^2^) = 0.102	H-atom parameters constrained
*S* = 1.04	*w* = 1/[σ^2^(*F*_o_^2^) + (0.0554*P*)^2^ + 0.3159*P*] where *P* = (*F*_o_^2^ + 2*F*_c_^2^)/3
1541 reflections	(Δ/σ)_max_ < 0.001
104 parameters	Δρ_max_ = 0.29 e Å^−^^3^
0 restraints	Δρ_min_ = −0.16 e Å^−^^3^

### Special details

**Table:**

Geometry. All e.s.d.'s (except the e.s.d. in the dihedral angle between two l.s. planes) are estimated using the full covariance matrix. The cell e.s.d.'s are taken into account individually in the estimation of e.s.d.'s in distances, angles and torsion angles; correlations between e.s.d.'s in cell parameters are only used when they are defined by crystal symmetry. An approximate (isotropic) treatment of cell e.s.d.'s is used for estimating e.s.d.'s involving l.s. planes.
Refinement. Refinement of *F*^2^ against ALL reflections. The weighted *R*-factor *wR* and goodness of fit *S* are based on *F*^2^, conventional *R*-factors *R* are based on *F*, with *F* set to zero for negative *F*^2^. The threshold expression of *F*^2^ > σ(*F*^2^) is used only for calculating *R*-factors(gt) *etc*. and is not relevant to the choice of reflections for refinement. *R*-factors based on *F*^2^ are statistically about twice as large as those based on *F*, and *R*- factors based on ALL data will be even larger.

### Fractional atomic coordinates and isotropic or equivalent isotropic displacement parameters (Å^2^)

**Table:**

	*x*	*y*	*z*	*U*_iso_*/*U*_eq_	
S1	0.49280 (7)	0.2500	0.16602 (12)	0.0352 (2)	
S2	0.70856 (7)	0.2500	−0.24173 (15)	0.0423 (2)	
O1	0.14361 (14)	0.45648 (5)	0.0468 (3)	0.0484 (4)	
C1	0.60102 (19)	0.29896 (6)	−0.2021 (4)	0.0364 (5)	
H1	0.6115	0.3262	−0.3011	0.044*	
C2	0.50486 (19)	0.29989 (6)	−0.0393 (3)	0.0307 (4)	
C3	0.40701 (18)	0.33990 (6)	−0.0143 (3)	0.0296 (4)	
C4	0.3199 (2)	0.34206 (7)	0.1786 (4)	0.0364 (5)	
H4	0.3213	0.3172	0.2931	0.044*	
C5	0.2311 (2)	0.38017 (7)	0.2059 (3)	0.0372 (5)	
H5	0.1746	0.3808	0.3381	0.045*	
C6	0.22660 (19)	0.41714 (7)	0.0376 (3)	0.0352 (5)	
C7	0.31115 (19)	0.41530 (7)	−0.1595 (4)	0.0395 (5)	
H7	0.3083	0.4399	−0.2753	0.047*	
C8	0.3984 (2)	0.37754 (7)	−0.1841 (4)	0.0371 (5)	
H8	0.4537	0.3769	−0.3180	0.044*	
C9	0.0549 (2)	0.45883 (8)	0.2462 (5)	0.0556 (6)	
H9A	0.0005	0.4301	0.2479	0.083*	
H9B	0.0002	0.4874	0.2311	0.083*	
H9C	0.1043	0.4606	0.3938	0.083*	

### Atomic displacement parameters (Å^2^)

**Table:**

	*U*^11^	*U*^22^	*U*^33^	*U*^12^	*U*^13^	*U*^23^
S1	0.0430 (4)	0.0328 (4)	0.0297 (4)	0.000	−0.0017 (3)	0.000
S2	0.0266 (4)	0.0405 (4)	0.0599 (5)	0.000	0.0101 (3)	0.000
O1	0.0487 (9)	0.0401 (8)	0.0563 (10)	0.0129 (7)	0.0112 (7)	0.0073 (7)
C1	0.0326 (10)	0.0306 (9)	0.0459 (12)	−0.0035 (8)	0.0042 (9)	0.0016 (8)
C2	0.0304 (9)	0.0287 (9)	0.0332 (11)	−0.0063 (8)	−0.0021 (8)	−0.0015 (7)
C3	0.0290 (10)	0.0274 (9)	0.0324 (10)	−0.0058 (8)	−0.0013 (8)	−0.0028 (7)
C4	0.0416 (11)	0.0314 (9)	0.0361 (11)	−0.0007 (8)	0.0067 (8)	0.0043 (8)
C5	0.0397 (11)	0.0362 (10)	0.0357 (11)	0.0002 (9)	0.0093 (9)	0.0000 (8)
C6	0.0333 (10)	0.0305 (10)	0.0420 (11)	−0.0001 (8)	−0.0015 (9)	−0.0015 (8)
C7	0.0421 (11)	0.0382 (10)	0.0381 (11)	0.0000 (9)	0.0019 (9)	0.0095 (9)
C8	0.0383 (11)	0.0397 (10)	0.0331 (11)	0.0004 (9)	0.0062 (8)	0.0030 (8)
C9	0.0548 (14)	0.0493 (13)	0.0627 (15)	0.0180 (11)	0.0168 (12)	0.0019 (12)

### Geometric parameters (Å, º)

**Table:**

S1—C2	1.7795 (18)	C4—C5	1.384 (3)
S1—C2^i^	1.7795 (18)	C4—H4	0.9300
S2—C1	1.7391 (19)	C5—C6	1.375 (3)
S2—C1^i^	1.7391 (19)	C5—H5	0.9300
O1—C6	1.365 (2)	C6—C7	1.389 (3)
O1—C9	1.426 (3)	C7—C8	1.365 (3)
C1—C2	1.328 (3)	C7—H7	0.9300
C1—H1	0.9300	C8—H8	0.9300
C2—C3	1.482 (3)	C9—H9A	0.9600
C3—C4	1.387 (3)	C9—H9B	0.9600
C3—C8	1.397 (3)	C9—H9C	0.9600
			
C2—S1—C2^i^	99.97 (12)	C4—C5—H5	120.0
C1—S2—C1^i^	100.54 (13)	O1—C6—C5	124.98 (18)
C6—O1—C9	116.92 (16)	O1—C6—C7	115.96 (17)
C2—C1—S2	124.07 (15)	C5—C6—C7	119.06 (18)
C2—C1—H1	118.0	C8—C7—C6	120.36 (18)
S2—C1—H1	118.0	C8—C7—H7	119.8
C1—C2—C3	124.65 (16)	C6—C7—H7	119.8
C1—C2—S1	118.03 (15)	C7—C8—C3	121.97 (18)
C3—C2—S1	117.32 (14)	C7—C8—H8	119.0
C4—C3—C8	116.60 (17)	C3—C8—H8	119.0
C4—C3—C2	121.94 (16)	O1—C9—H9A	109.5
C8—C3—C2	121.46 (16)	O1—C9—H9B	109.5
C5—C4—C3	121.98 (17)	H9A—C9—H9B	109.5
C5—C4—H4	119.0	O1—C9—H9C	109.5
C3—C4—H4	119.0	H9A—C9—H9C	109.5
C6—C5—C4	120.00 (18)	H9B—C9—H9C	109.5
C6—C5—H5	120.0		
			
C1^i^—S2—C1—C2	39.0 (2)	C3—C4—C5—C6	0.6 (3)
S2—C1—C2—C3	−175.75 (14)	C9—O1—C6—C5	−0.4 (3)
S2—C1—C2—S1	4.9 (2)	C9—O1—C6—C7	179.31 (19)
C2^i^—S1—C2—C1	−48.1 (2)	C4—C5—C6—O1	−179.75 (18)
C2^i^—S1—C2—C3	132.49 (11)	C4—C5—C6—C7	0.6 (3)
C1—C2—C3—C4	−171.26 (19)	O1—C6—C7—C8	179.64 (18)
S1—C2—C3—C4	8.1 (2)	C5—C6—C7—C8	−0.7 (3)
C1—C2—C3—C8	8.5 (3)	C6—C7—C8—C3	−0.5 (3)
S1—C2—C3—C8	−172.18 (14)	C4—C3—C8—C7	1.6 (3)
C8—C3—C4—C5	−1.7 (3)	C2—C3—C8—C7	−178.16 (18)
C2—C3—C4—C5	178.07 (17)		

Symmetry code: (i) *x*, −*y*+1/2, *z*.
